# Osmium and OsO _x_ nanoparticles: an overview of syntheses and applications

**DOI:** 10.12688/openreseurope.14595.2

**Published:** 2022-08-01

**Authors:** Jonathan Quinson

**Affiliations:** 1 Chemistry, University of Copenhagen, Copenhagen, Denmark; 2 Biochemical and Chemical Engineering, Aarhus University, Aarhus, Denmark

**Keywords:** Osmium, Nanoparticles, Nanomaterials, Catalysis, Synthesis, Clusters, Colloids, Applications

## Abstract

Precious metal nanoparticles are key for a range of applications ranging from catalysis and sensing to medicine. While gold (Au), silver (Ag), platinum (Pt), palladium (Pd) or ruthenium (Ru) nanoparticles have been widely studied, other precious metals are less investigated. Osmium (Os) is one of the least studied of the precious metals. However, Os nanoparticles are interesting materials since they present unique features compared to other precious metals and Os nanomaterials have been reported to be useful for a range of applications, catalysis or sensing for instance. With the increasing availability of advanced characterization techniques, investigating the properties of relatively small Os nanoparticles and clusters has become easier and it can be expected that our knowledge on Os nanomaterials will increase in the coming years. This review aims to give an overview on Os and Os oxide materials syntheses and applications.

## Plain language summary

Precious metals are rare and expensive materials. However, they present unique properties that make them relevant for many applications, for instance in catalysis and medicine. Numerous studies focus on gold (Au), silver (Ag), platinum (Pt), palladium (Pd), ruthenium (Ru) or rhodium (Rh). Recently, iridium (Ir) is gaining interest for use in developing more sustainable energy conversion. The interest on precious metals extend to less studied materials like osmium (Os). In order to make the most of every atom of these metals, developing nanomaterials like clusters and nanoparticles is a rewarding strategy. With the increasing work and knowledge gained on precious metals in general, it is expected that some of these less studied materials will be opening new opportunities. This review provides an overview of the work performed to date on osmium nanoparticles.

## Introduction

Precious metals, such as gold (Au), silver (Ag), platinum (Pt), palladium (Pd), ruthenium (Ru), rhodium (Rh) or iridium (Ir), are critical and expensive materials. Nevertheless, they play a key role in catalysis ^1, 2^, water/air treatment ^3^ and medicine ^4^. Molecules comprising one of few atoms or precious metals stabilized by ligands in complexes have been largely investigated for use as catalysts or in medical applications ^5^. More recently, nanomaterials made of several hundreds or thousands of metal atoms have been investigated for their unique properties ^6^ relevant for medicine ^4^, chemical synthesis and catalysis ^7^, sensing ^8^, water/air purification ^9^, optics ^10^, electronics ^11^, building and construction ^12^, to name only a few examples.

**Figure 1.**
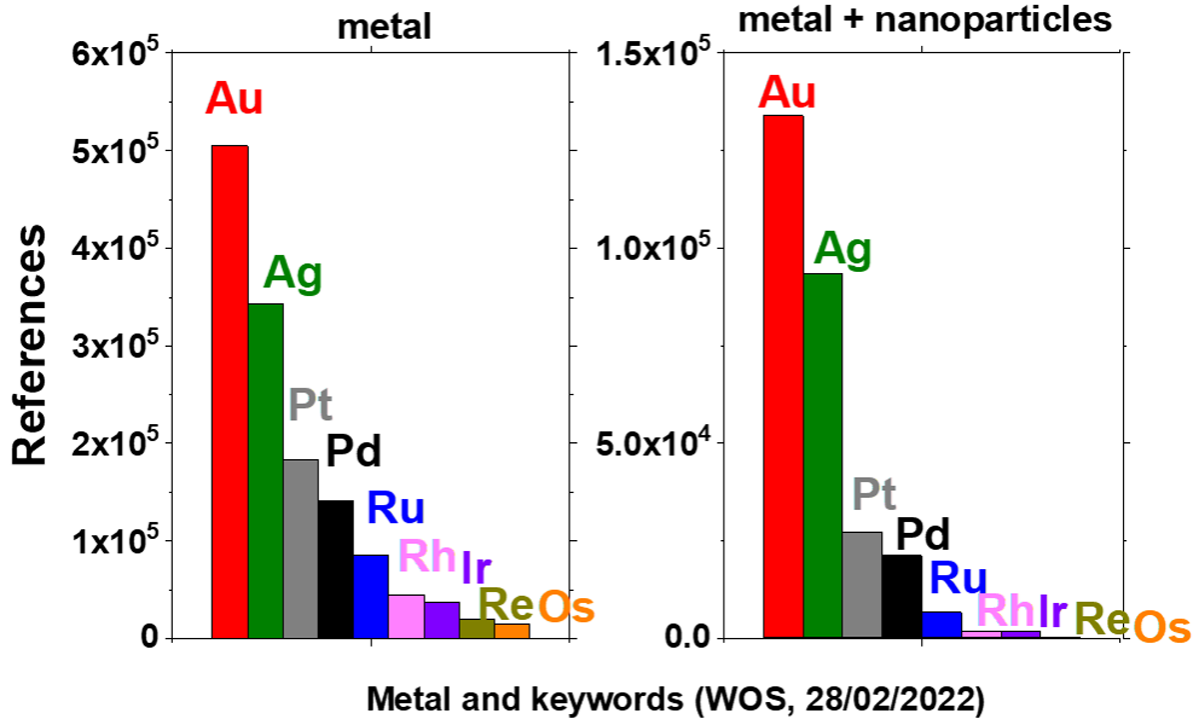
Number of references returned for searches on the Web of Science database with different keywords, ‘metal’ or ‘metal+nanoparticles’, where ‘metal’ is gold (Au), silver (Ag), platinum (Pt), palladium (Pd), ruthenium (Ru), rhodium (Rh), iridium (Ir), rhenium (Re) or osmium (Os), as indicated.

For precious metals, a trend in the literature is to focus on Au, Ag, Pt, Pd, Ru or Rh nanoparticles and nanomaterials. Figure 1 shows the results from a search on the Web of Science (WOS) database (Clarivate Analytics) with different keywords including ‘metal’ and ‘nanoparticles’. These results show the number of references returned for different combination of keywords and metals. A clear trend is that the least studied precious metals are iridium (Ir), rhenium (Re) and osmium (Os) - assuming that the number of references returned for each search gives an indication of the importance of the related research area. This can be explained by the fact that these metals are among the least available on Earth ^13^. The focus here is on the least studied material: Os.

**Figure 2.**
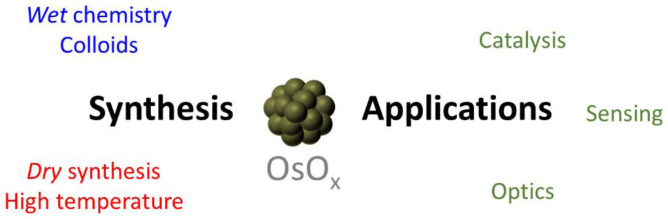
Overview, aim and scope of this review into Osmium oxide (OsO _x_).

Os is the densest metal and has been mainly studied for its mechanical properties ^14, 15^. However, Os nanomaterials also show promising features for applications in catalysis and medicine ^16^. There is, to the best of our knowledge, no review on Os nanoparticles. Os nanoparticles are partially covered in a very recent review witch mainly focuses on Ru and Rh and catalytic applications ^2^. In addition to its natural scarcity, the relatively limited amount of work on Os nanomaterials can be inferred to the typically smaller size (<2 nm) for Os nanoparticles compared to other precious metals, for most syntheses reported ^17^. This small size makes the nanomaterials challenging to characterize. In addition, the relative limited number of reports on Os can be related to the fact that Os easily get oxidized to OsO _x_ materials such as OsO _4_, a highly toxic compound ^18^. Nevertheless, OsO _4_ has been commonly used as a staining agent in microscopy ^19^ and in catalysis ^20^. Os complexes and clusters have been used as model systems over many years, for instance in the work of Professor Gates ^21^. Based on the knowledge already available on Os complexes, it is expected than the interest and knowledge on Os nanomaterials will grow in the coming years. This review proposes an overview of Os nanoparticles syntheses and applications. Rather than a detailed discussion of selected work, the aim is here to give a broad view of work reported to date on Os nanoparticles, as illustrated in Figure 2.

## Discussion/analysis of the recent literature

### Formation mechanism

It is expected that understanding the formation mechanism(s) of nanomaterials will be a key to develop more controlled syntheses ^22^. This in turn will lead to nanomaterials designed with tuned properties to best match the requirements for a given application. Certainly, materials like Au nanoparticles have been intensively investigated and a relatively clear picture of the nanoparticle formation has been proposed ^23, 24^. Nevertheless, several questions remain to understand and control how atoms of metal in a complex form larger nanomaterials, e.g. even for the case of well-studied metals like Pt ^25^. It can be observed that metals for which the synthesis can easily be followed by simple techniques, such as ultraviolet-visible spectroscopy (UV-vis) for Au or Pt, have been more intensively studied. It is therefore tempting to explain the relatively limited knowledge on Os nanoparticles by the challenging characterization of the related materials. Importantly, the risk of forming the toxic OsO _4_^18^ is also a bottleneck in the investigation of Os nanoparticles compared to Au or Pt.

A specific feature of Os nanomaterials is to lead to relatively small (<2 nm, see Table 1) nanostructures, regardless of the synthesis approach used. For this size range, most characterization techniques, until recently, are not easily implemented to evaluate size, shape and structure or to follow the formation mechanism of Os nanomaterials. Recently, using a combination of complementary *in situ* X-ray diffraction (XRD), quick X-ray absorption fine-structure (QXAFS) and X-ray photoelectron spectroscopy (XPS) performed at synchrotron facilities, the formation at high temperature of PdOs nanoparticles from [Pd(NH _3_) _4_][OsCl _6_] was studied ^44^. Such advanced studies are much needed to better understand the formation of nanomaterials but remain scarce for Os and Os based materials. Another example is the use of X-ray total scattering with pair distribution function (PDF) analysis, also requiring access to synchrotron facilities, where Os _x_Cl _y_ intermediates structures were suggested for the formation of Os nanoparticles in a colloidal approach ^42^. Despite a relatively poor understanding on how Os nanomaterials form, and few reports focusing on the formation mechanism of Os nanoparticles, a range of successful syntheses have been reported and are illustrated in Table 1.

**Table 1.** Examples of literature on osmium oxide (OsO _x_) nanoparticles synthesis and applications.

Ref	Date	Precursor	Method, solvent, support, additives, conditions	Use	Size / nm
*Dry syntheses*					
17	1979	OsO _4_	Impregnation	cyclohexene hydrogenation	< 1
26	2007	[Os _3_CO _10_(NCMe) _2_]	Pyrolysis (acetone) - carbon nanotubes	design of Os nanotubes	< 3
27	2008	Os metal carbonyls	Pyrolysis – SiO _2_	-	1-10
28	2012	Os(C _5_H5) _2_	Atomic layer deposition	-	Films and nanoparticles
29	2013	Os(COD) (cyclooctatetraene)	Impregnation (pentane) SiO _2_ – H _2_ reduction	alkanes hydrogenolysis	1.1
30	2015	Home made Os ^II^ complex	Electron beam induced synthesis	-	1.5-50
31	2017	Home made Os ^II^ complex	Microwave Laser	-	1-50
32	2019	Home made Os ^II^ complex	Electron beam induced synthesis	temperature effect on nucleation	< 2
*Wet chemical syntheses*					
17	1979	OsO _4_ 0.4 mM	Alcohol + water + PVP reflux	cyclohexene hydrogenation	< 1
33	2005	OsCl _3_ 19 mM	ethylene glycol, NaOH, 160 °C, 3 h	-	0.6-1.8
34	2010	OsCl _3_ 2 mM	H _2_O, HEPES, EPPS, PIPES, MES 180 °C, 1-3 h, autoclave	aerobic oxidation of alcohols to aldehydes	1.6
35	2013	OsCl _3_	H _2_O, AA 95 °C, 1.5 h	SERS	1.0-1.5
36	2014	OsO _4_ 0.9 mM	H _2_O, NaOH CTAB, 2,7-DHN RT, 30 min	catalysis, SERS (nanoparticles or chains)	1-3
37	2014	OsO _4_ 0.7 mM	DNA, TBABH _4_ RT, 10 h	cyclohexene hydrogenation SERS	1-3 Shape control
38	2014	OsO _4_ 0.9 mM	SDS, NaBH _4_ RT, 30 min	SERS and KMnO _4_ decomposition (nanoparticles or chains)	1.2-2.5
39	2018	OsCl _3_	THF, LiEt _3_BH RT, 2 h	benzyl alcohol oxidation	1.3
40	2020	K _2_OsCl _6_ 0.2 mM	H _2_O, NaBH _4_, heparin RT, 1.5 h	sensing	1.8
41	2020	Os(acac) _3_ 12 mM OsCl _3_ 40 mM	Ethylene glycol, PVP 200 °C, 12 h H _2_O, NaBH _4_, RT	structure control ( *hcp* vs. *fcc*)	1-2
42 43	2022	OsCl _3_ H _2_OsCl _6_	2.5-100 mM methanol, ethanol, H _2_O 85 °C, 6 h – 1 week	-	1-2

AA: ascorbic acid; COD: 1,5-cyclooctadiene; CTAB: cetyltrimethylammonium bromide; EPPS : 3-[4-(2-hydroxyethyl)-1-piperazinyl] propanesulfonic acid; 2,7-DHN: 2,7-dihydroxynaphthalene; HEPES: (4-(2-hydroxyethyl)-1-piperazineethanesulfonic acid; MES: 2-ethanesulfonic acid; PIPES: piperazine-N,N′-bis(2-ethanesulfonic acid); PVP: polyvinylpyrrolidone; RT: room temperature; SERS: surface-enhanced raman spectroscopy; SDS: sodium dodecyl sulfate; TBABH _4_: tetrabutylammonium borohydride; *fcc*: face-centered cubic; *hcp*: hexagonal close packed.

### Dry syntheses

As opposed to wet chemical syntheses detailed below, where the formation of Os nanoparticles proceeds in the liquid phase, a range of high temperature dry syntheses are reported for Os nanoparticles. Typically a support material is needed to stabilize the nanoparticles ^2^. An overview of different syntheses is proposed and an example of synthesis is the thermal decomposition of Os precursors ^45^. Pyrolysis leads to different nanoparticle size depending on the ligand structures of the precursors ^27^ and needs to be performed at relatively high temperature, e.g. 300 °C, when the precursor is an Os carbonyl complex ^26^. Hydrogen (H _2_) reduction is also an option ^29^. Magnetron sputtering has been reported for Os films ^46^. Alternative methods include wet incipient impregnation ^47^, freeze drying ^48^ or atomic layer deposition (ALD) of Os films and particles ^28^. However, in this last approach and in this specific study, where osmocene and molecular oxygen were used as precursors, the challenge was the formation of highly toxic OsO _4_ at high temperature.

### Wet chemical syntheses

Wet chemical or colloidal syntheses are very popular synthetic approaches to obtain a range of nanomaterials directly relevant for multiple applications ^1, 7, 49^. The formation of nanoparticles proceeds in a solvent via the reduction of a metal complex in an oxidized state in the presence of a reducing agent ^50^, followed by the growth of the nanoparticles ^51^. In most cases, the syntheses do not require a support material. This is an advantage to truly investigate support and loading-related effects in catalysis since the nanoparticle formation and control over the nanoparticle size and other properties is independent of the presence of a support ^52^. Typically, the syntheses are performed in presence of a range of additives like surfactants to stabilize the nanoparticles.

Os nanoparticles can be obtained from a variety of solvents and reducing agents summarized in Table 1. Surfactants typically added for the synthesis are for example heparin ^40^, (4-(2-hydroxyethyl)-1-piperazineethanesulfonic acid) (HEPES) ^34^, 3-[4-(2-hydroxyethyl)-1-piperazinyl] propanesulfonic acid (EPPS) ^34^, piperazine-N,N′-bis(2-ethanesulfonic acid) (PIPES) ^34^, 2-ethanesulfonic acid (MES) ^34^, polyvinylpyrrolidone (PVP) ^17^, sodium dodecyl sulfate (SDS) ^38^, DNA ^37, 53^, sodium citrate ^54^ and various precursors are suitable to obtain Os nanoparticles. OsCl _3_ remains a common precursor. OsCl _3_ can be reduced at room temperature (RT) using a strong reducing agent like LiEt _3_BH (superhydride) in tetrahydrofuran (THF) ^39^. The nanoparticles are *circa* 1.3±0.2 nm. NaBH _4_ is also a suitable reducing agents for RT synthesis ^38^. In a range of other syntheses, temperature between 80 to 200 °C are typically used depending on the solvent selected, see Table 1. In methanol-water in presence of PVP, sub-nanometer nanoparticles are obtained ^17^. Ionic liquids are also suitable to obtain nanoparticles for instance from the metal carbonyl precursor Os _3_(CO) _12_^55, 56^. The reaction of OsO _4_ in aqueous solution of cetyltrimethylammonium bromide (CTAB), 2,7-dihydroxynaphthalene (2,7-DHN) and NaOH, leads to nanoparticles *circa* 1-3 nm ^36^. Adjusting the concentration of CTAB, different morphologies made of individual nanoparticles, chain-like or aggregated clusters were obtained. Chains of Os nanoclusters are also obtained using ascorbic acid (AA) as a reducing and capping agent in an aqueous medium to lead to nanoparticles in the size range 1-1.5 nm with properties suitable for surface-enhanced raman spectroscopy (SERS) ^35^.

**Figure 3.**
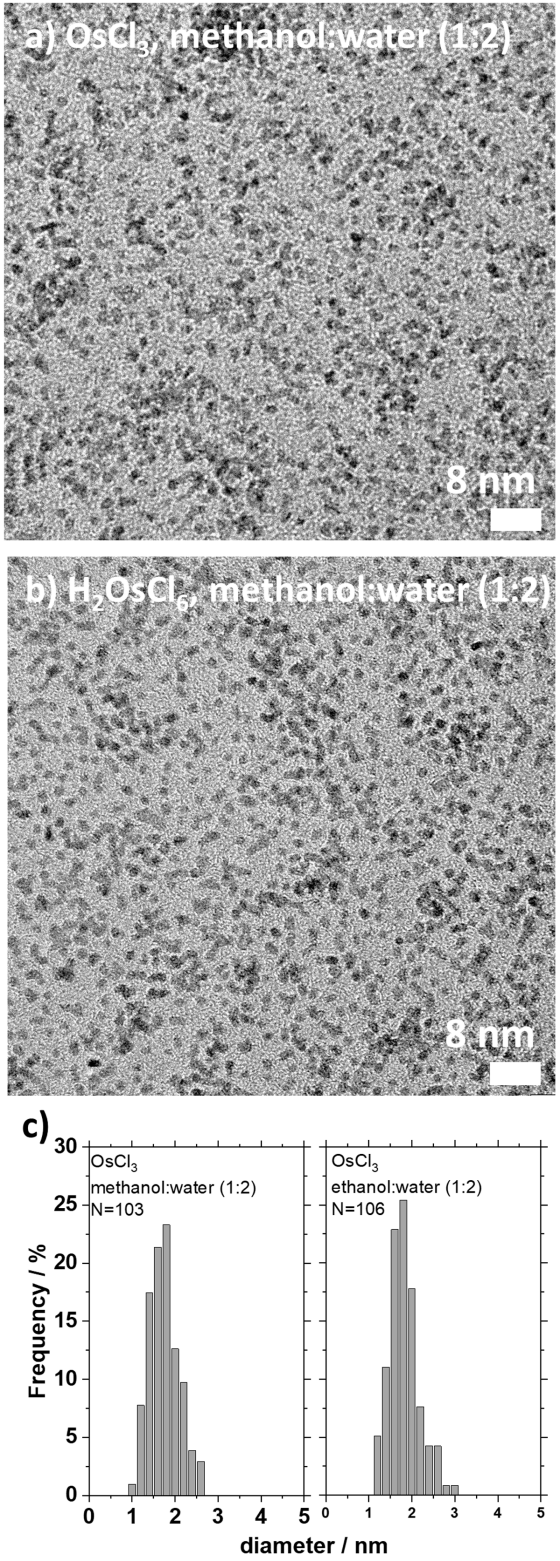
Example of small size Osmium (Os) nanoparticles. ( **a**– **b**) transmission electron microscope (TEM) micrographs of Os nanoparticles obtained using water (66 volume %) and methanol (33 volume %) and 100 mM of ( **a**) OsCl _3_ and ( **b**) H _2_OsCl _6_ as precursors after a one-week reaction at 85 °C. The size analysis ( **c**) suggests that the nanoparticles are ( **a**) 1.6±0.4 nm and (b) 1.7±0.3 nm. Reproduced from 42 with permission from the Beilstein-Institut.

Os nanoparticles are typically small (<2 nm) across different syntheses ^34^. This therefore questions the actual need to stabilize the small nanoparticles. Developing surfactant-free colloidal syntheses, although it is challenging, is possible ^57^. Surfactant-free nanoparticles with a more accessible surface to reactants are directly relevant for catalysis. Surfactant-free nanoparticles are also more simply modified, for instance with dedicated ligands and molecules towards bio-medical applications. Examples of surfactant-free nanoparticles include the polyol synthesis ^33^, typically performed in alkaline ethylene glycol, or recently reported mono-alcohol synthesis ^1^, performed in alkaline methanol or ethanol. In the latter case, it was actually shown that high precursor concentrations up to 100 mM ^43^ and even without the need for a base ^42^, leads to the formation of small size <3 nm Os nanoparticles, see Figure 3. Such small size nanoparticles were obtained across a large parametric study investigating the time of synthesis from hours to weeks, nature and concentration of precursors, solvent composition and reducing agent (methanol or ethanol) as well as base concentration.

**Figure 4.**
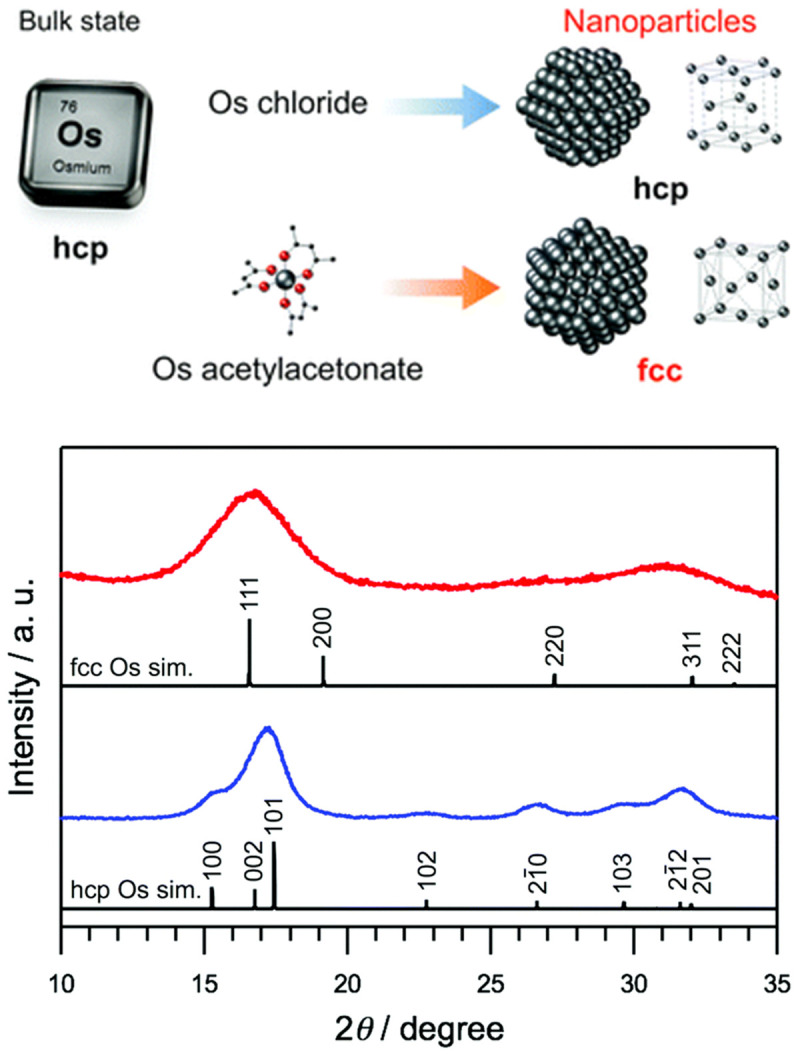
Tuning osmium (Os) nanoparticle structures by controlled synthesis. (Top) Schematic of the formation of face-centered cubic (fcc) or hexagonal close packed (hcp) Os nanoparticles depending on the precursor used. (Bottom) Synchrotron X-ray diffraction (XRD) patterns of Os nanoparticles synthesized using the Os(acac) _3_ complexes (red) and (blue) OsCl _3_, and the simulations of fcc (upper black) and hcp Os (lower black). Reproduced from 41 with permission from the Royal Society of Chemistry.

A recent work showed that face-centered cubic ( *fcc)* nanoparticles instead of the expected hexagonal close packed ( *hcp)* structure could be obtained by careful choice of the precursor, reducing agent and solvent, see illustration in Figure 4. - Iridium is the neighbor transition metal of Os and adopts the *fcc* structure. The difference in total energy between the *hcp* and *fcc* structures of Os is expected to be small and so it should be possible to obtain *fcc* Os nanoparticles. In presence of ethylene glycol and PVP using Os acetylacetonate (Os(acac) _3_), *fcc* nanoparticles were obtained whereas *hcp* nanoparticles were obtained with OsCl _3 _in water using NaBH _4_ as reducing agent ^41^. The change in structure is attributed to the role of the acac ligand that can stabilize the nearest-neighbor Os–Os bond length (ca. 2.67 Å) in a close-packed plane of Os, that is close to the O–O length (2.74–2.93 Å) of the acac ligand. This leads to nanoparticles with a different crystal structure. The question of whether or not this would happen is the synthesis was performed under exactly the same conditions (same precursor concentration, reducing agents and solvents) but only changing the precursor remains open. Size selected nanoparticles were obtained in a two-phase (water-toluene) approach from OsO _4_ and tetrabutylammonium borohydride (TBABH _4_): 1±0.2 nm, 10–30 nm, 22±2 nm and 31±3 nm nanoparticles were synthesized by changing the concentration ratio of the metal precursor and the amount of reductant ^37^.

### Os complexes and clusters

Compared with Os nanoparticles, Os complexes have been more studied to date ^20, 58– 60^. For instance, Os metal carbonyl complexes have been widely investigated ^61^. The group of Professor Gates intensively studied Os _n_ clusters ^62, 63^. In particular Os carbonyls clusters were widely investigated on various support like gold ^64^, MoS _2_^61^, zeolite ^65^, MgO ^62, 63, 66, 67^ with a focus on conversion from complexes to clusters. Carbonyls clusters were investigated by ^129^Xe nuclear magnetic resonance (NMR), where [HOs _3_(CO) _11_] ^-^ or [H _3_Os _4_(CO) _12_] ^-^ were found to formed in zeolites ^65^ and [Os _3_(CO) _12_] on MgO ^66^.

**Figure 5.**
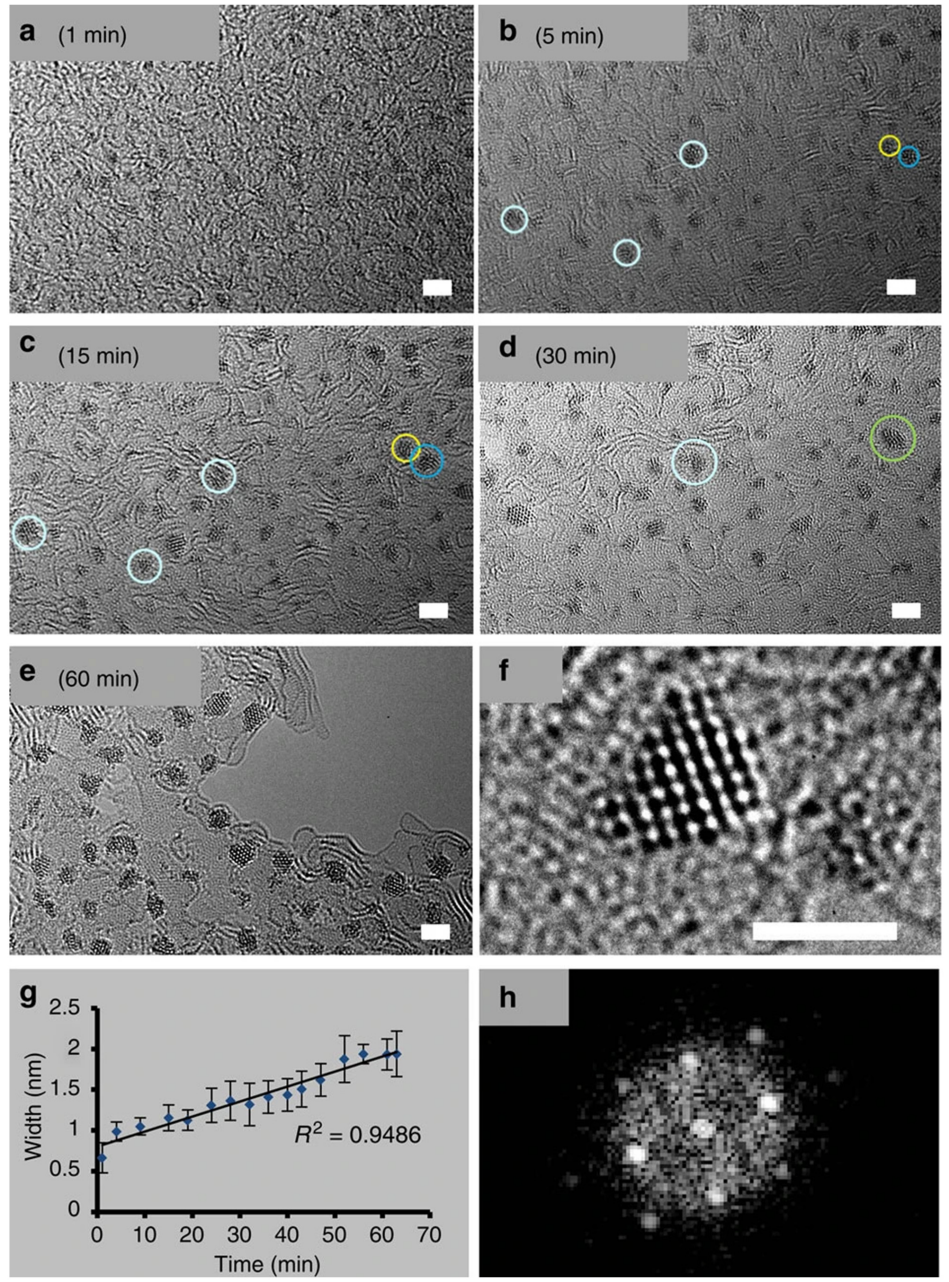
Os nanoparticles as model system to study the formation and stability of nanomaterials. ( **a**– **d**) Migration of small Os clusters and their coalescence (e.g. clusters in yellow and dark blue circles merge to give crystal in green circle) over a period 1–30 min; scale bars, 2 nm. ( **e**) Nanocrystals after 60 min. ( **f**) Example of an Os crystal of ca. 1.5 nm, formed after 30 min of irradiation, scale bar, 1.5 nm. ( **g**) Width of the clusters/crystals versus time. ( **h**) Fast Fourier transform analysis of the nanocrystal shown in **f**. Reproduced from 68 with permission from Springer Nature.

Barry *et al*. used Os atoms and complexes as their model system for various studies, e.g. to build up 3D nanocrystals to observe, study and quantify crystal growth at the atomic scale controlled in real time ^68, 69^, see the illustration in Figure 5. The experiments were conducted using the electron beam of a transmission electron microscope (TEM) and a micelle-stabilized complex of [Os( *p*-cymene)(1,2-dicarba- *closo*-dodecarborane-1,2-dithiolate)] ^30^. The same precursor under microwave irradiation leads to supported Os nanoparticles *circa* 1 nm in diameter ^31^. Os was used to show the temperature dependent nucleation and growth kinetics of precious metal nanocrystals supported on silicon nitride by aberration corrected TEM ^32^. Barry *et al.* for that purpose used homemade Os complexes in that study. The growth rate was found to be dependent on the temperature ( *circa* 2.5 times faster at 100 °C than at 20 °C). No effect of the temperature on the crystal structure of the nanocrystals was observed, ’’although the sizes of the crystals (<2 nm) and the very small number of atoms per crystal render clear elucidation of the structures extremely difficult’ *’* ^32^. The challenging characterization of Os nanoparticles by routine equipment indeed remains a bottleneck.

### Applications

Os nanomaterials found applications in a wide range of fields and a broad overview is proposed here.

***Chemical synthesis.*** Recent reports suggest that Os nanomaterials might have specific properties for hydrogenation reactions compared to other precious metals ^70^. Os based materials have been used as catalysts for dihydroxylation ^71^, cyclohexene hydrogenation ^17, 37^, citral hydrogenation ^72^. Other reactions include oxidation of benzyl alcohol with relatively low yield compared to Ir ^39^ or reduction of 4-nitro aniline ^36^. Using HEPES protected nanoparticles, the conversion of *p*-methylbenzylalcohol ^34^, *p*-methoxybenzylalcohol ^34^, *p*-bromobenzylalcohol ^34^, 3-phenyl-2-propanol ^34^, 3-phenyl-2-propenol (cinnamylalkohol) ^34^, 1-phenylethanol ^34^, lead in all cases to the aldehyde or ketones in relatively high yield. On silica doped with zirconium, Os nanoparticles show reactivity for hydrogenation and hydrogenolysis/hydrocracking of tetralin from aqueous K _2_[OsCl _6_] ^47^. A high Os content displays weak hydrogenation activity but very good hydrogenolysis/hydrocracking activity. Os nanomaterials are also suitable for the synthesis of various 1,2-cis-diols ^73^, 1,2/3-triols synthesis from the allylic hydroperoxides ^74^, *syn*-dihydroxylation of alkenes ^75^, reduction of *p*-nitroaniline into *p*-phenyldiamine ^76^, oxidations reaction ^39^, CO oxidation ^77^, ammonia synthesis ^78^ or Fischer–Tropsch synthesis ^79^. Under aerobic condition, oxidation of activated, unactivated and heteroatom containing alcohols to carbonyl compounds lead to high activity and selectivity even under mild conditions ^80^.

**Figure 6.**
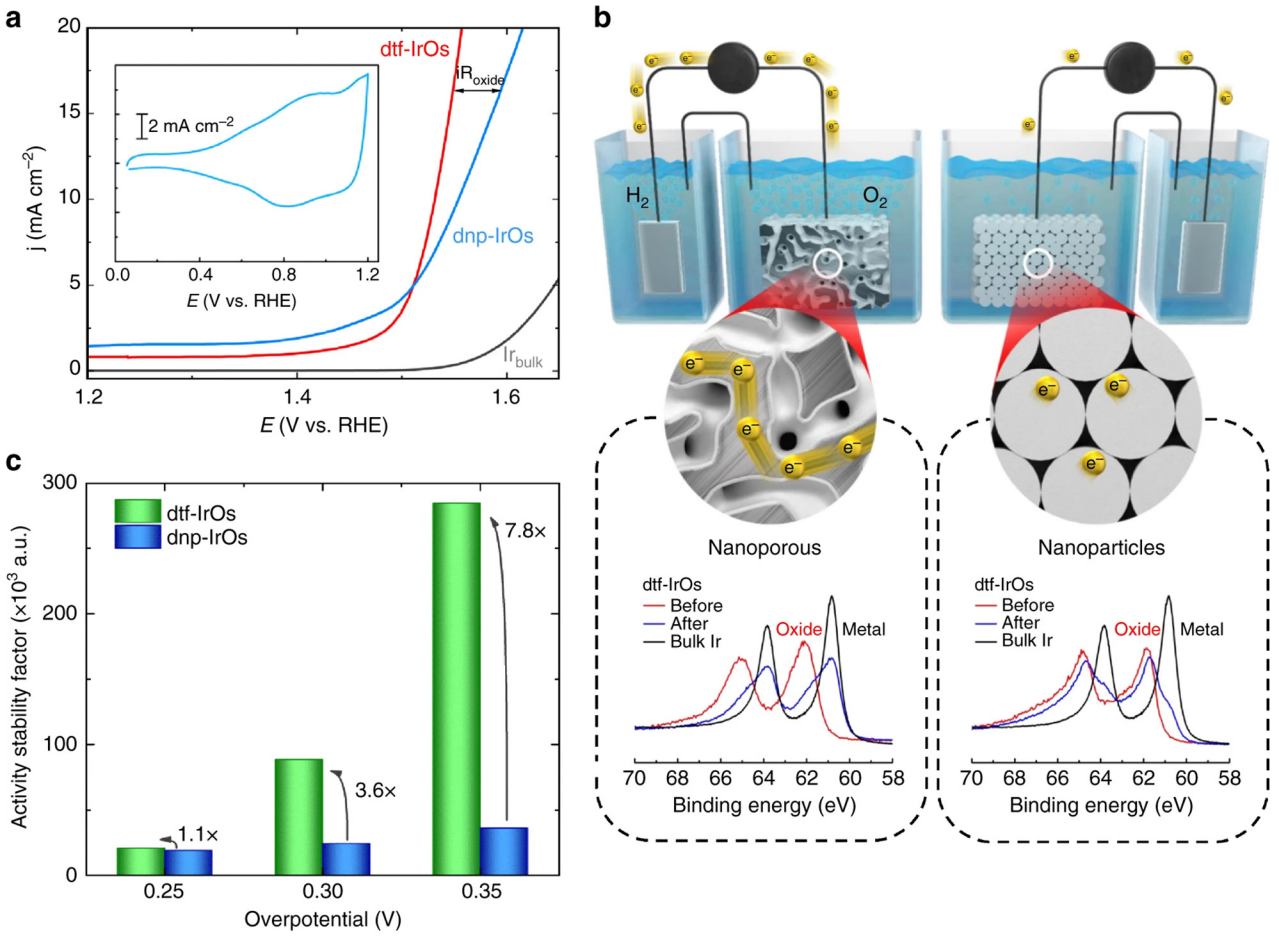
Electrochemical properties of Osmium (Os)-based nanomaterials: Activity-conductivity relationships in de-alloyed thin-film and nanoparticles. ( **a**) Comparison between oxygen evolution reaction (OER) polarization curves for polycrystalline Iridium (Ir), de-alloyed thin film (dtf) Ir _25_Os _75_ and de-alloyed nanoparticles (dnp) Ir _50_Os _50_, indicating that conductivity limitations are observed for dnp-Ir _50_Os _50_ at higher current densities (denoted as iR _oxide_). Inset shows the corresponding cyclic voltamograph. ( **b**) X-ray photoelectron spectroscopy (XPS) sputter etching experiments demonstrating that the dtf-Ir _25_Os _75 _consists of an IrO _x_ shell with Ir-metallic core, in contrast to dnp-Ir _50_Os _50_ that consists entirely of IrO _x_. Schematic illustrates the impact of multiple oxide-oxide interfaces (present on dnp-Ir _50_Os _50_ electrodes) on conductivity. ( **c**) Change in activity-stability factor values with overpotential for dtf-Ir _25_Os _75_ and dnp-Ir _50_Os _50 _highlighting the importance of balancing activity-stability-conductivity properties of oxide materials for the OER. Reproduced from 86 with permission from Springer Nature.

***6.2.5.2. Electrochemistry.*** OsO _x_ materials have been shown to be suitable for a range of electrochemical reactions including hydrogen evolution reaction (HER) ^81^, oxygen reduction reaction (ORR) ^82^ or as direct borohydride polymer electrolyte membrane fuel cell anodes ^83^. Freeze drying was used to obtain Os/Si nanowires and the corresponding nanoparticles by etching the Si nanowires ^48^. In this comparative study with Rh, Pt, Pd, Re, Ru, Au or Ag nanocomposites, Os was found to give the higher activity for the HER, a small onset potential of -25 mV and long term stability ^48^. Using magnetron sputtered Os it was found that the high activity of OsO _x_ for the HER in acidic media was correlated with poor stability ^84^. Nanoparticles based on Os are easy to de-alloy, e.g. Pt _2_Os to from quasi core-shell Os@Pt for ORR in acidic media ^85^. This property can be used to develop high surface area materials by de-alloying, e.g. to develop improved porous-electrodes for the oxygen evolution reaction (OER), see Figure 6 ^86^. Os itself is expected to show very high activity for the OER but suffer from poor stability ^84^.

**Figure 7.**
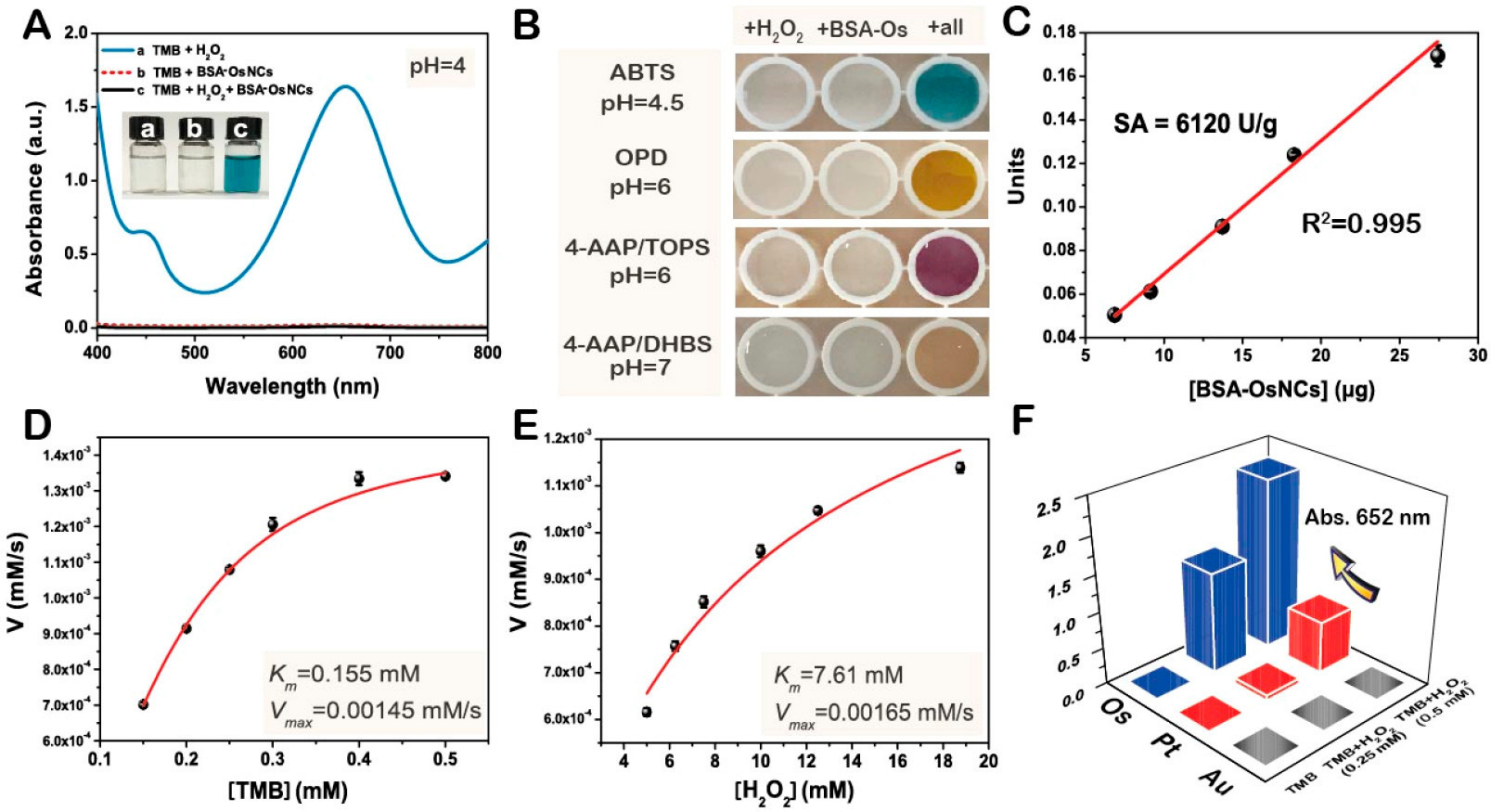
Osmium (Os) nanoparticles for sensing. ( **A**) Ultra-violet/visible light (UV-vis)spectra of 3,3′,5,5′-tetramethylbenzidine (TMB) + H _2_O _2_ (0.25 mM), TMB + bovine serum albumin (BSA)–Os nanoparticles (Os content = 1 mM), and TMB + H _2_O _2_ (0.25 mM) + BSA–Os nanoparticles (Os content = 1 mM). Inset: Corresponding photographs. ( **B**) Corresponding photographs of some peroxidase substrates catalyzed by BSA–Os nanoparticles in the presence of H _2_O _2_: substrate + H _2_O _2_, substrate + BSA–Os nanoparticles, and substrate + H _2_O _2_ + BSA–Os NCs. ( **C**) Specific activity of BSA–Os nanoparticles. Steady-state kinetic assay of BSA–Os nanoparticles toward ( **D**) TMB and ( **E**) H _2_O _2_. ( **F**) A _652nm_ of TMB, TMB + H _2_O _2_ (0.25 mM), and TMB + H _2_O _2_ (0.5 mM) catalyzed by BSA–Au nanoparticles (Au content = 1 mM), BSA–Pt nanoparticles (Pt content = 1 mM), and BSA–Os nanoparticles (Os content = 1 mM). Reprinted with permission from 87. Copyright 2022 American Chemical Society.

***6.2.5.3. Other applications.*** Os nanomaterials are less studied than other precious metals for medical applications ^60^ or pollution management ^37^. However, Os nanoparticles found recent applications in sensing. Os nanoparticles protected by heparin as the protecting/stabilizing agent were used as a heparinase sensor ^40^. Bovine serum albumin is an efficient protective shell to give Os nanoparticles an antifouling property regarding various ions (e.g., Hg ^2+,^ Ag ^+^, Pb ^2+^, I ^−^, Cr ^6+^, Cu ^2+^, Ce ^3+^, S ^2−^, etc.), saline (0−2 M), or protein (0−100 mg/mL) conditions. A colorimetric sensor was developed for H _2_O _2_ detection with improved properties compared to Au or Pt based sensors ^87^, see the illustration in Figure 7. Other examples include glucose and pyruvic acid detections ^54^, folic acid detection ^88^ colorimetric sensors for heavy metal ions discrimination (Cu ^2+,^ Ag ^+^, Cd ^2+,^ Hg ^2+,^ and Pb ^2+^) ^89^. Os nanoparticles also show SERS properties ^36, 37, 76^.

### Theory

Less work has been performed on Os nanoparticles than Ir ^90^ or Pt ^25^ nanoparticles but some theoretical work can be found in the literature ^70, 91– 93^. For instance, Os was suggested to be a suitable catalysts for ammonia production ^94^. While being less investigated than Ir, Os _xn_ clusters were studied by density functional theory (DFT) for instance in light of their interaction with MgO for n=4,5 ^91^. By analogy with what is available for Ir or Pt, it can be expected that theoretical work will be valuable to clarify why small size nanoparticles are easily obtained, which might be related to the formation of ‘magic number’ nanoparticles with specific sizes ^95^ and/or sintering resistance properties ^96^. Equaly theoretical work could be relevant to explore further the properties of Os based nanomaterials, in particular towards improved stability.

### Os in multi-metallic nanomaterials

In addition to the examples already mentioned above, for instance in Figure 6, various alloyed nanoparticles have been reported such as IrOs ^14^, PtOs ^97^, OsB _2_^98^, in particular for their improved mechanical properties. PdOs nanoparticles were reported as catalyst for carbon nanotube synthesis ^26^. Other examples include NiOs _4_ reported for the improved hydrogenation of cinnamaldehyde ^99^, PtOs for the methanol oxidation reaction ^100^, CuOs for the methanol oxidation reaction and ORR ^101^ or OsTe nanorods for cancer therapy ^102^.

## Discussion

A range of Os nanomaterials can easily be obtained by various syntheses methods, see Table 1. In particular, a range of surfactant-free syntheses are well documented and are expected to lead to Os nanoparticles with improved properties in fields of applications like catalysis and sensing. However, the characterization of the small (<2 nm) nanoparticles obtained in most cases remains one of the bottlenecks in the study of Os nanomaterials. Relatively complex characterization techniques (not routine) are needed such as high-angle annular dark-field scanning transmission electron microscopy (HAADF-STEM) ^41^, or synchrotron based measurements ^41^, see Figure 4. For instance, X-ray diffraction technique will lead to large Bragg peaks for such small nanoparticles and most TEM equipment will not easily characterize such small nanomaterials. Also, the size range around 1 nm is at the limit of most small angle X-ray scattering (SAXS) equipment.

However, recent progress in the characterization of nanomaterials ^103^, and in particular the increasing availability of high resolution TEM or techniques like X-ray total scattering with pair distribution function (PDF) analysis ^42, 104^, are well suited to characterized nanocrystals. Recent advances in these techniques are expected to bring new insights into Os nanomaterial formation. The knowledge gained will be the key for improving syntheses of nanomaterials towards more functional materials. There is a regain of interest on Ir and Ir oxide nanoparticles, in great part due to high expectations on Ir as a potential catalyst for OER ^105^. Ir and Os chemistry are relatively similar in the sense that they both easily lead to small size nanoparticles and clusters. This makes them ideal candidates to study nanoparticle formation and to focus on nucleation phenomena since the nanoparticle growth is moderate.

In addition, the Os materials obtained are relevant for a range of applications. In particular, high expectations are on new or improved applications in catalysis and medicine. An example of emerging opportunity is for instance the possibility to investigate the different catalytic properties of *fcc* or *hcp* Os nanoparticles, see Figure 4, largely unexplored to date.

Finally, it is expected that the interest on iridium ^90^ will trigger increasing interest in Os nanoparticles, which in turn will enable further exploration of Os chemistry. However, for long term applications recycling is an important issue to address ^106^, in particular in light of the relatively poor stability of Os in application like electrochemical energy conversion ^84^. In this respect, the role and stability of Os in increasingly studied bimetallic ^86^ and even high entropy alloys ^107^ is also an opening area of research.

## Conclusions

Despite a limited knowledge on the actual formation mechanism of Os nanoparticles, several approaches lead to simple syntheses of Os nanoparticles. The very small size *circa* 1–2 nm of most Os nanoparticles suggests that a range of reported syntheses probably can be simplified, *e.g*. avoiding the use of any surfactants or high temperature. Relatively high concentration of precursors can be used and still lead to small size nanoparticles which is a promising feature for future scaling. The obtained OsO _x_ nanoparticles already proved to be relevant for a wide variety of applications in particular as active materials in catalysis or as templating agents. (Re)Emerging areas of application include chemical synthesis ^79^, sensing ^89^ or medical applications ^60^.

## Data availability

No data are associated with this article

## Ethics and consent

Ethical approval and consent were not required.
