# Supplementary material for: Osmium and OsO x nanoparticles: an overview of syntheses and applications
Source: Open Res Eur. 2022 Aug 1;2:39. Originally published 2022 Mar 21. [Version 2] doi: 10.12688/openreseurope.14595.2 (PMC10446100; doi:10.12688/openreseurope.14595.2)
Supplement: Peer review history

# Peer review: Reviewer response for version 2

**DOI:** 10.21956/openreseurope.16220.r29820

**Published:** 2022-08-02

**Authors:** Michael Nolan

**Affiliations:** 1 Tyndall National Institute, University College Cork, Cork, Ireland

The author has given very good responses to the original review comments and improved the original submission.

 I am very happy to recommend acceptance of this interesting submission.

Is the review written in accessible language?

Yes

Are all factual statements correct and adequately supported by citations?

Yes

Are the conclusions drawn appropriate in the context of the current research literature?

Yes

Is the topic of the review discussed comprehensively in the context of the current literature?

Partly

Reviewer Expertise:

First principles simulations, nanoparticles and nanostructures, surface chemistry

I confirm that I have read this submission and believe that I have an appropriate level of expertise to confirm that it is of an acceptable scientific standard.

# Peer review: Reviewer response for version 1

**DOI:** 10.21956/openreseurope.15760.r29489

**Published:** 2022-07-25

**Authors:** Shaojun Guo

**Affiliations:** 1 School of Materials Science and Engineering, Peking University, Beijing, China

**Comments to the Authors**

 In this work, Quinson and co-authors reviewed the recent advances in the syntheses and applications of Os and Os-based nanoparticles. They first summarized several important synthesis methods of Os-based nanomaterials. Then, several key applications of Os-based catalysts were introduced, such as chemical synthesis, electrocatalysis and medical applications, etc *.* This review is logical and well-organized, and provides a valuable reference for the future design of advanced Os-based nanomaterials. In view of the timely summary/highlight and importance of this work, I recommend its indexing, only after a minor revision to address the following concerns.

1. There are too many keywords.
2. The authors proposed that Os-based materials present unique features compared to other precious metals. What are these unique features?
3.  It is pointed out that the nanoparticles with very small sizes are difficult to characterize. However, I think this is no longer challenging, because many technologies have been used to characterize their structures, even some atomically dispersed catalysts, such as atomic-resolution aberration-corrected scanning transmission electron microscopy.
4. Some possible trends and challenges for future advanced research directions should be included at the end of this manuscript.
5. In **Figure 4**, there are two-phase structures for Os nanoparticles ( *fcc* and *hcp*). The influence of two kinds of Os on electrocatalysis should be mentioned.

Is the review written in accessible language?

Yes

Are all factual statements correct and adequately supported by citations?

Yes

Are the conclusions drawn appropriate in the context of the current research literature?

Yes

Is the topic of the review discussed comprehensively in the context of the current literature?

Yes

Reviewer Expertise:

Electrocatalysis

I confirm that I have read this submission and believe that I have an appropriate level of expertise to confirm that it is of an acceptable scientific standard.

# Response

**Published:** 2022-07-29

**Authors:** Jonathan Quinson

**Affiliations:** Aarhus University, Denmark

I would like to thank the reviewer for his/her/their time to review the work and valuable comments. Answers to the comments are below:

1. The number of 8 keywords is following the recommended numbers by the Open Research Europe guidelines: https://open-research-europe.ec.europa.eu/for-authors/article-guidelines/science-technology-and-medicine/reviews/#keywords.
2.  I understand that the reviewer refers here to the wording in the abstract. The wording is kept general to fit the abstract requirements. These unique features are somehow specified in the rest of the sentence ‘catalysis or sensing for instance’ and detailed all along the review. It is also stressed later that Os is one of the densest metal known. These unique features can be summed up as the different physico-chemical properties highlighted in the review, such as small sizes, and the relevance for specific applications detailed in the *Applications* section, such as catalysis, medical applications, etc.
3. The reviewer is right, and certainly how small a nanoparticle must be to start being ‘challenging’ to characterize depends on many factors. In particular, the type of equipment available to a research group will be a critical factor. However, it can be safely considered that the resolution of most transmission electron microscope (TEM) starts being limited for nanomaterials less than 1-2 nm. And certainly, high resolution TEM (HRTEM) and more advanced scanning transmission electron microscopy (STEM) are (fortunately) increasingly routine equipment. The increasingly availability of these characterization techniques is indeed expected to bring increasingly new insights into atomically structured materials.
  The comments made here were maybe to be understood considering the historical development of precious metal nanomaterial studies. As reported in *Figure 1*, Ag and Ag nanomaterials have been the most studied. This is, I believe, in great part due to their well-defined plasmonic properties which makes it possible to study the nanomaterials (and even estimate size) by simple techniques such as UV-vis. Also, a range of synthetic methods allow to form nanomaterials with a size above 10 nm, relatively easy to characterize with most TEM equipment. In contrast Pt, Pd tend to lead to smaller nanoparticles, say 2-10 nm, across various synthetic approaches, and so are less simple to characterize. Further comes Ru, Rh, Ir and Os which tend to lead to even smaller nanoparticles. The rough relationship ‘larger size correlates to larger number of research studies’ might be in part due to the facility to study relatively larger nanoparticles.
  I agree with the reviewer that the bottleneck related to characterization is maybe less challenging to date, but it still accounts for the fact that there are fewer studies on Ir or Os nanoparticles. Hence, there are high expectations on new discoveries on the related materials, now that this bottleneck is at least partially alleviated, as stressed in the second paragraph of the *Discussion* section.     
4.  A generic statement can be found in the *Discussion* section. This section has been re-shaped into four paragraphs and not two, to best stress future directions of research on both fundamental aspects of nanoparticle formation (and the related characterization) and more applications. An example of an emerging new opportunity is given for catalysis, considering the comment (5) from the reviewer.
5.  The reviewer is right, and this has been stressed better now in the revised version of the work: ‘’ An example of emerging opportunity is for instance the possibility to investigate the different catalytic properties of fcc or hcp Os nanoparticles, see *Figure 4*, largely unexplored to date ‘’ has been added.

# Peer review: Reviewer response for version 1

**DOI:** 10.21956/openreseurope.15760.r29491

**Published:** 2022-06-20

**Authors:** Michael Nolan

**Affiliations:** 1 Tyndall National Institute, University College Cork, Cork, Ireland

In this submission the authors presents a review of Os and OsOx nanoparticles focussing on their synthesis and applications.

 Overall, the review is interesting and brings together work on a metal that receives much less attention than other precious metals.

 I have some comments that I hope can enhance the submission.

1. Some comments on aspects of the language:
  Introduction "along the years" can be replaced by "over many years" (or similar)
  Page 4 "is to easily lead to relatively small"
2. Introduction: Is Os the densest metal or element?
3. Table 1 caption: add "synthesis" at the end of the caption text. Is there a chemical formula for the "Home-made" complex?
4. Author mentions a challenge with the ALD of Os nanoparticles, that is formation of toxic OsO4. Did the ALD use an oxygen source? A H2 plasma could be one option to reduce to Os metal.
5. Page 7 mentions how different Os precursors can produce different structured nanoparticles - do the original papers have any discussion on the origin of this difference. For me stating "role of ligands that can stabilise a specific facet" is not sufficient.
6. I may have missed it, but given that Os appears to make small (2 nm) nanoparticles, can the author make some discussion on the origin of this?
7. The theory part is probably much too short to be of real value. The author can either remove it or expand to give some more details.
8. In multimetallic systems, is the role of Os described in the literature? What value is there in making Os alloys for chemistry?

Is the review written in accessible language?

Yes

Are all factual statements correct and adequately supported by citations?

Yes

Are the conclusions drawn appropriate in the context of the current research literature?

Yes

Is the topic of the review discussed comprehensively in the context of the current literature?

Partly

Reviewer Expertise:

First principles simulations, nanoparticles and nanostructures, surface chemistry

I confirm that I have read this submission and believe that I have an appropriate level of expertise to confirm that it is of an acceptable scientific standard, however I have significant reservations, as outlined above.

# Response

**Published:** 2022-07-29

**Authors:** Jonathan Quinson

**Affiliations:** Aarhus University, Denmark

I would like to thank the reviewer for his/her/their time to review the work and the comments provided to improve the work. Here are the answers to the points above:

1. The language-related matters have been improved.
2. Os is the densest metal (Girolami, G.S. Osmium weighs in. *Nat Chem* 2012, 4, 954-954, DOI:10.1038/nchem.1479). Also, it is among the densest naturally occurring elements.
3. The caption of Table 1 has been corrected in the new version by adding: ‘’Table 1. Examples of literature on osmium oxide (OsO _x_) nanoparticle synthesis and applications’’
4. There is certainly a chemical forumal for the ''Home-made'' complex, given in the main text, but in light of the overview provided, we do not wish to complicate the Table and hope that interested readers will refer to the relevant literature.
  These complexes are for instance:
  [Os(η6-p-cym)(1,2-dicarba-closo-dodecarborane-1,2-dithiolato)]
  [Os(η6-p-cym)(1,2-dicarba-closo-dodecarborane-1,2-dithiolato)(triphenylphosphine)
5. The comment from the reviewer was: *''Author mentions a challenge with the ALD of Os nanoparticles, that is formation of toxic OsO _4_. Did the ALD use an oxygen source? A H _2_ plasma could be one option to reduce to Os metal.''* This is a fair point raised by the reviewer. In the specific study referred to where ALD was used, oxygen was used as co-precursor (Hämäläinen J, Sajavaara T, Puukilainen E, et al.: Atomic Layer Deposition of Osmium. *Chem Mater. *2012;24(1):55–60, DOI: 10.1021/cm201795s). I am not familiar enough myself with the ALD requirements but the suggestion of the reviewer makes sense. I guess this is one of the many aspects of Os chemistry still to be explored.
6. The comment from the reviewer was: *''Page 7 mentions how different Os precursors can produce different structured nanoparticles - do the original papers have any discussion on the origin of this difference. For me stating "role of ligands that can stabilise a specific facet" is not sufficient.'' *The original paper (Wakisaka T, Kusada K, Yamamoto T, *et al*.: Discovery of face-centred cubic Os nanoparticles. *Chem Commun*. 2020;56(3):372–374, 31808775, DOI: 10.1039/c9cc09192k) certainly does discuss this. The discussion has now be completed: by adding: ''A recent work showed that face-centered cubic ( *fcc*) nanoparticles instead of the expected hexagonal close packed ( *hcp*) structure could be obtained by careful choice of the precursor, reducing agent and solvent, see illustration in *Figure 4*. Iridium is the neighbour transition metal of Os and adopts the *fcc* structure. The difference in total energy between the *hcp* and *fcc* structures of Os is expected to be small and so it should be possible to obtain *fcc *Os nanoparticles. In presence of ethylene glycol and PVP using Os acetylacetonate (Os(acac) _3_), *fcc* nanoparticles were obtained whereas *hcp *nanoparticles were obtained with OsCl _3_ in water using NaBH _4_ as reducing agent [41]. The change in structure is attributed to the role of the acac ligand that can stabilize the nearest-neighbour Os–Os bond length (ca. 2.67 Å) in a close-packed plane of Os, that is close to the O–O length (2.74–2.93 Å) of the acac ligand. This leads to nanoparticles with a different crystal structure.''
7.  To the best of my knowledge, there is no definite framework to explain why Os nanoparticles are easily obtained with small sizes, although it is a very clear trend in the literature (Hirai H, Nakao Y, Toshima N: Preparation of Colloidal Transition Metals in Polymers by Reduction with Alcohols or Ethers. *J Macromol Sci Chem. *1979; A13(6):727–750, DOI: 10.1080/00222337908056685). Just like for other transition metals, it is very likely that ‘magic number’ nanoparticles are stable and can be obtained. The open question is the driving force probably differing for different metals that seem to lead to some of the magic clusters more than others (smaller one or larger ones depending on the metal, see Reference / comments 42 in Watzky, M.A.; Finke, R.G. Nanocluster size-control and "magic number" investigations, experimental tests of the "living-metal polymer" concept and of mechanism-based size-control predictions leading to the syntheses of iridium(0) nanoclusters centering about four sequential magic numbers. *Chem. Mater.* 1997, 9, 3083-3095, DOI:10.1021/cm9704387). The best analogy to propose an explanation to the observed small size of Os nanoparticles is probably to compare with the neighbor element Iridium, for which it has been suggested that some small structures are so stable that the nanoparticles will be resistant to sintering (Lu, J.; Aydin, C.; Browning, N.D.; Wang, L.C.; Gates, B.C. Sinter-Resistant Catalysts: Supported Iridium Nanoclusters with Intrinsically Limited Sizes. *Catal. Lett.* 2012, 142, 1445-1451, DOI:10.1007/s10562-012-0928-8). As far as I know, this property has not been fully explored and explained for osmium. This discussion comes relevant together with the comment (8) from the reviewer.
8. The reviewer is right that the theory part is relatively scarce. Especially when comparing with what can be found on Iridium (Quinson, J. Iridium and IrO _x _nanoparticles: an overview and review of syntheses and applications. *Adv Colloid and Interface Sci.* 2022, 303, 102643, DOI:10.1016/j.cis.2022.102643). In light of this ‘lack’ of theoretical studies, this section is now including comments along the line of the answer made on comment (7) from the reviewer: lack of explanation of the small size of the Os nanoparticles observed across the literature. This now reads as follows: ''Less work has been performed on Os nanoparticles than Ir [90: Quinson, J. Iridium and IrO _x_ nanoparticles: an overview and review of syntheses and applications. *Adv Colloid Interface Sci*. 2022, 303, 102643, DOI:10.1016/j.cis.2022.102643] or Pt [25: Quinson J, Jensen KMØ: From platinum atoms in molecules to colloidal nanoparticles: A review on reduction, nucleation and growth mechanisms. *Adv Colloid Interface Sci.* 2020;286:102300. 33166723, DOI: 10.1016/j.cis.2020.102300] nanoparticles but some theoretical work can be found in the literature [70, 91-93] . For instance, Os was suggested to be a suitable catalyst for ammonia production [94]. While being less investigated than Ir, Os _n _clusters were studied by density functional theory (DFT) for instance in light of their interaction with MgO for n=4,5 [91]. By analogy with what is available for Ir or Pt, it can be expected that theoretical work will be valuable to clarify why small size nanoparticles are easily obtained, which might be related to the formation of ‘magic number’ nanoparticles with specific sizes [95: Watzky, M.A.; Finke, R.G. Nanocluster size-control and "magic number" investigations, experimental tests of the "living-metal polymer" concept and of mechanism-based size-control predictions leading to the syntheses of iridium(0) nanoclusters centering about four sequential magic numbers. *Chem. Mater.* 1997, 9, 3083-3095, DOI:10.1021/cm9704387] and/or sintering resistance properties [96: Lu, J.; Aydin, C.; Browning, N.D.; Wang, L.C.; Gates, B.C. Sinter-Resistant Catalysts: Supported Iridium Nanoclusters with Intrinsically Limited Sizes. *Catal. Lett.* 2012, 142, 1445-1451, DOI:10.1007/s10562-012-0928-8]. Equally theoretical work could be relevant to explore further the properties of Os-based nanomaterials, in particular towards improved stability."
9. Comment from the reviewer: ' *'In multimetallic systems, is the role of Os described in the literature? What value is there in making Os alloys for chemistry?''* This is once more a very open question. Most studies will not consider Os as an alloying element due to the risk of Os leaching, for instance for the OER (Danilovic N, Subbaraman R, Chang KC, *et al*.: Activity-Stability Trends for the Oxygen Evolution Reaction on Monometallic Oxides in Acidic Environments. *J Phys Chem Lett.* 2014;5(14):2474–2478. 26277818. DOI: 10.1021/jz501061n). Also forming porous materials by Os leaching seems an expensive strategy, although it has shown to bring useful features such as higher OER activity in some cases (Kim YT, Lopes PP, Park SA, *et al*.: Balancing activity, stability and conductivity of nanoporous core-shell iridium/iridium oxide oxygen evolution catalysts. *Nat Commun.* 2017;8(1):1449. 29129907. DOI: 10.1038/s41467-017-01734-7 5682288). The lack of consideration for Os can also come from the related toxicity and risk to form OsO _4_. Finally, a generally small number of reports, and even fewer theoretical reports as highlighted in the answer to comment (7), accounts for the scarcity of rational arguments to use Os in general, and in bi/multi metallic in particular. Nevertheless, the activity of Os for catalytic applications, e.g. for the OER, is very promising if the stability issues can be addressed. In this respect, the range of opportunities offered by developing Os based materials is still to be explored. In this regard, the emerging interest around high entropy alloys (Miracle, D.B.; Senkov, O.N. A critical review of high entropy alloys and related concepts. *Acta Materialia* 2017, 122, 448-511, DOI:10.1016/j.actamat.2016.08.081) might bring new highlights in the properties of Os-based nanomaterials and this is now stressed more in the manuscript with the relevant new references here mentioned: Finally, it is expected that the interest on iridium [90: J. Iridium and IrO _x_ nanoparticles: an overview and review of syntheses and applications. *Adv Colloid Interface Sci.* 2022, 303, 102643, DOI:10.1016/j.cis.2022.102643] will trigger increasing interest in Os nanoparticles, which in turn will enable further exploration of Os chemistry. However, for long term applications recycling is an important issue to address [106], in particular in light of the relatively poor stability of Os in application like electrochemical energy conversion- [84]. In this respect, the role and stability of Os in increasingly studied bimetallic [86: Kim YT, Lopes PP, Park SA, *et al.*: Balancing activity, stability and conductivity of nanoporous core-shell iridium/iridium oxide oxygen evolution catalysts. *Nat Commun. *2017;8(1):1449. 29129907, DOI:10.1038/s41467-017-01734-7 5682288] and even high entropy alloys [107: Miracle, D.B.; Senkov, O.N. A critical review of high entropy alloys and related concepts. *Acta Materialia* 2017, 122, 448-511, DOI:10.1016/j.actamat.2016.08.081] is also an opening area of research.
